# Approaching a collaborative research agenda for health systems performance in circumpolar regions

**DOI:** 10.3402/ijch.v72i0.21474

**Published:** 2013-08-14

**Authors:** Susan Chatwood, Jessica Bytautas, Anthea Darychuk, Peter Bjerregaard, Adalsteinn Brown, Donald Cole, Howard Hu, Micheal Jong, Malcolm King, Siv Kvernmo, Jeremy Veillard

**Affiliations:** 1 Dalla Lana School of Public Health, University of Toronto, Toronto, Canada; 2 Institute for Circumpolar Health Research, Yellowknife, Canada; 3 Institute of Health Policy, Management and Evaluation, University of Toronto, Toronto, Canada; 4 University of Southern Denmark, Copenhagen, Denmark; 5 Centre for Health Research in Greenland, Nuuk, Greenland; 6 Labrador-Grenfell Regional Health Authority, Happy Valley-Goose Bay, Canada; 7 Memorial University of Newfoundland, Happy Valley-Goose Bay, Canada; 8 Simon Fraser University, Burnaby, Canada; 9 University of Tromsø, Tromsø, Norway; 10 Canadian Institute for Health Information, Toronto, Canada

Health care in Canada's north and circumpolar regions faces considerable challenges with the remote and widely dispersed population, harsh environmental conditions, and human resource challenges. Despite per capita expenditures that are among the highest in the world, health outcomes continue to lag behind the rest of Canada, and health disparities between the Indigenous and non-Indigenous populations within the north persist. While improving the health of northerners requires addressing underlying social determinants, transforming the health care system holds promise for health improvements in the short- and medium-term.

The evidence required to inform a northern-focused and relevant transformation of health care systems remains to be generated and applied. This seminar set out to identify priority areas for a research initiative that will address systems challenges and engage decision-makers in these jurisdictions. The overarching objectives of the seminar were to explore the priority areas for health systems research in circumpolar regions, and to propose how we might best maximize our current resources, and facilitate partnerships for the advancement of a common agenda.

## Process

The seminar highlighted emerging issues through facilitated panels with representatives of sectors engaged in the circumpolar health system, including clinicians, administrators, policy-makers, and Indigenous groups. Geographic coverage spanned the United States (Alaska), Canada (NWT, Nunavut, Labrador), Norway, Greenland, Denmark, and Iceland. The seminar program is provided in the Appendix.

With an eye to summarizing key findings from the day and making recommendations to advance future research activities in the field, a panel of experts was brought together to serve as a “jury” and reflect in committee. While the approach was cursory, the intent was to highlight the main points made throughout the seminar and provide a summary that would guide the development of research collaborations and programs.

## Objectives

The seminar provided an opportunity to network and advance a shared research agenda that would inform and compliment mandates of policy-makers, systems managers, Indigenous leaders, and researchers. Seminar questions were formulated to focus the discussion, and highlight priority areas for health systems research, data needs, and best practices for research partnerships.

### Seminar questions

1. What are the existing health systems challenges and resulting priority areas for health systems research in circumpolar regions?
2. What do we need from a scholarly perspective to maximize uptake of data and evidence currently available?
3. What are the best practices for health research partnerships that engage academic partners, community sectors, health authorities, and government?

### Findings

Seminar findings under each of the workshop questions are highlighted below, and recommendations for an approach to research collaboration that advances systems improvements in circumpolar regions are highlighted. In general, these recommendations are directed to researchers, funders, and decision-makers. There was no opportunity to refine the recommendations by target group or collaborations within the timeframe of the workshop. However, this step is recommended as questions are developed and plans for knowledge translation are made.

Question 1: *What are the existing health systems challenges and resulting priority areas for health systems research in circumpolar regions?*

The panel presentations provided an informed perspective and introduced the context of delivering health services in circumpolar regions. Common issues raised reflected what has been described in the literature. Recognized were the challenges inherent in delivering services in remote and scattered populations, in harsh environments and, to marginalized Indigenous populations, along with a lack of training for management and delivery, and substantial financial resources devoted to health care. As experiences of health systems responses to these same challenges were put forth, a strong sense of resilience emerged through the description of health system reforms in Greenland, responses to economic collapse in Iceland, repatriation of mental health services to Sami control in Norway, and systems performance frameworks and programs responsive to Indigenous populations in Alaska.

The resulting recommendations on priority areas for health systems research were not only specific to systems performance within models familiar to national and international comparisons, but many comments arose that raised common issues that relate to the context and values underlying health and wellness, specifically in Indigenous populations and to broader definitions of health and health systems that recognized the underlying determinants of health. As a result, there was considerable emphasis placed on the need to understand underlying context and values, and to take whole of government and whole of society approaches. In addition to the broader context for health systems, specific research areas were highlighted relating to systems operations in remote regions, mental health, prevalent diseases, aging populations, and social determinants of health.

**Key recommendations**

Avoid imposing a narrow paradigm of what “health system” means. Look to multi-disciplinary and cross-sector approaches.

Take into consideration stewardship frameworks to better articulate the scope of activities as they pertain to systems and health-status improvements.

Articulate the values and context with respect to health and wellbeing, especially as it pertains to indigenous and non-indigenous peoples.

Build on Indigenous models for health care delivery in circumpolar regions.

Develop models and measures that inform practices that bridge “physical” and “mental” health care systems.

Highlight systems’ responses to providing equal access regardless of residence. Specific examples were related to the use of telemedicine, EMRs, medical evacuations, and standards for medical visits (by provider and by patient) where provided.

Respond to increasing demand for health services due to population growth, and aging population, mental health, injury burden, and an increase in chronic diseases.

Learn from international comparisons to understand models of care. Create international comparisons between regions with shared values and contexts.

Question 2: *What do we need from a scholarly perspective to maximize uptake of data and evidence currently available?*

In addition to the utilization of current evidence and data held by national, territorial and northern regional authorities, the need for community-specific and timely population health databases was highlighted. Specifically, the potential of electronic medical records was raised. The on-going work within the Government of the Northwest Territories, Labrador Grenfell Health, and Southcentral Foundation was highlighted.

The need for participatory action and community-based research methods and data were highlighted. These methods were seen to best capture local values and utilize knowledge held in Indigenous sectors. They also tend to lead to community capacity development. The need to examine the ways in which traditional healing methods can be integrated with the biomedical model of health service delivery was raised as a specific area where such data could be informative. Early successes in the development of community-based approaches in Nunavut at the Qaujigiartiit Health Research Centre demonstrated the utility of these methods in identifying and engaging at-risk youth in mental health program development.

An overarching need to base research design on an integrated performance-oriented framework, with multi-sector partnerships that inform systems improvements and management practice in the north was highlighted. The time constraints of the workshop did not allow for further discussion on specifics related to uptake of the data and evidence currently available. However, the need to create robust research and evaluation programs as new initiatives are implemented was highlighted, thus ensuring that health sectors and the broader community can learn from the implementation of promising practices.

**Recommendations**

Begin with the fundamentals, including the need for baseline data and comparable governance practices/policies.

Recognition of broader interpretations of the system context and diversity of value systems that may require unique approaches to data and indicators.

Consider health system stewardship functions as data are organized. Be responsive to the need for data sharing across health systems and community sectors, and to the requirements of data scope and integrity.

Recognize the potential of community-based partnerships, health authorities, and data sources (e.g. traditional knowledge, EMRs, territorial databases).

Ensure database development notes mechanisms for dissemination of analysis outputs, notes implications for promising practices, and provides access to decision-makers for management decisions and system improvements.

Question 3: *What are the best practices for health research partnerships that engage academic partners, community sectors, health authorities, and government?*

The strengths of academic, government, and community partnerships were seen to be formative to relevant and applied research that would in-turn, direct health systems improvements. Good partnerships ensure research questions are well designed and responsive to the circumpolar context, values, and community. These partnerships also create the foundation for research programs that are oriented to health systems performance and for frameworks that guide researchers, policy-makers, and management.

A common theme arose regarding the lack of capacity to sustain partnerships and conduct research across sectors in northern jurisdictions. While many funding programs support collaboration, resource allocations and policies to sustain northern engagement in research are often lacking. It was recognized that partnerships that not only bring in expertise, but also build capacity through training and education programs among northerners, should be supported through long-term collaborations.

The current memorandum of understanding between the University of Toronto and the Institute for Circumpolar Health Research and the appointment of northern-based faculty provides a sustainable framework and policies to support northern-based graduate students and research. Labrador has similar capacity with faculty on site and agreements between the health authority and Memorial University. Greenland is also building capacity for northern-based activities with partnerships between the government, University of Greenland, University of Southern Denmark and the Centre for Health Research in Greenland. Regions such as Alaska and countries such as Iceland have well-developed academic infrastructure and capacity for health research.

**Recommendations**

Expand the research agenda across sectors responsible for health and wellness, and recognize academic and Indigenous knowledge bases.

Northern sectors and academic partners need to work collaboratively, set priorities, and focus on long-term research agendas.

Priorities need to be set through collaborative mechanisms that engage and recognize the roles and contributions of researchers, decision-makers, managers, and clinicians.

Through academic, community, and government partnerships, build research capacity in terms of data, networks, scholars, and policy-makers trained in the use of evidence.

In circumpolar regions where northern-based academic and community research centres are in early development, recognize the importance of research models that support sustained capacity for northern-based activities and research policy development.

## Conclusion

The workshop set out to address specific questions that would help direct a collaborative research agenda for health systems performance in circumpolar regions. While elements of the questions were addressed at a refined level, much of the discussion focused on broader elements regarding the context for health systems in circumpolar regions and underlying values for health. Presentations provided narratives of health sectors and individuals’ experiences as they interacted within health systems and adapted to the unique features of the circumpolar context.

There are early indications that shared values and contexts exist between circumpolar regions. These values are rooted in Indigenous traditions that are holistic and value contributions of the broader society. Further study is required to fully understand these values and contexts, and present these in a framework that will support international comparisons and systems improvements within the circumpolar context.

For many, the concept of health systems research as a field that informs decision-making and management was a newer concept. Participants made the distinction as to what health systems as an entity was, as opposed to health outcomes and the understanding of diseases and their distributions (a more familiar field of study). Within the discussions on health systems, performance frameworks were raised as appropriate lenses to further explore the resilience of health systems in circumpolar environments, and to apply lessons to systems design and management. Which frameworks most readily apply to the circumpolar context, requires further study and discussion among partners.

Built-in mechanisms for reciprocal education and capacity building were seen to be a critical component of research partnerships. The imbalance between partners’ knowledge bases in research and management theory, northern health policy, and cultural elements can be bridged, and capacity built, through reciprocal educational initiatives. Education is a dynamic tool that can close the capacity gap between partners and facilitate a comprehensive and sustainable research program that improves systems management and operations.

In summary, the seminar highlighted the elements of the shared context, challenges and resulting resilience in circumpolar health systems. A cursory list of priorities and approaches for partnerships were highlighted. Further collaboration is required to formulate mechanisms for partnerships, articulate common values and goals for health systems, and implement frameworks to guide the study and improvements of circumpolar health systems in a circumpolar context.

*Post-workshop note: Following the workshop, the Canadian Institutes for Health Research announced an award under the Community-Based Primary Health Care team grant program, entitled “Transforming primary health care in remote northern communities: the Circumpolar Health Systems Innovation Team”. This is a 5-year research program under the leadership of Kue Young, involving teams from NWT, Nunavut, Labrador and Greenland and several Canadian universities. Many team partners include workshop participants who will aim to carry forward workshop recommendations*.

## Appendix

**Seminar Program**

Speakers and panelists

*Opening remarks*

Dr. Donald Cole, Director, Institute for Global Health Equity and Innovation, Dalla Lana School of Public Health, University of Toronto, Toronto, Canada

*Introduction*

Dr. Kue Young, Professor, Dalla Lana School of Public Health, University of Toronto, Toronto, Canada

*Panel on circumpolar health systems challenges and responses*

*Moderator*

Dr. Adalsteinn Brown, Director, Institute of Health Policy, Management and Evaluation, University of Toronto, Toronto, Canada

*Panelists*

Ms. Monita O'Connor, Assistant Deputy Minister of Operations, Government of Nunavut, Iqaluit, Canada

Ms. Helga H. Bjarnadóttir, Director, Department of Economics, The National University Hospital of Iceland, Reykjavik, Iceland

Ms. Debbie DeLancey, Deputy Minister, Department of Health and Social Services, Government of the Northwest Territories, Yellowknife, Canada

*Panel on indigenous adaptations in circumpolar regions*

*Moderator*

Ms. Susan Chatwood, Executive and Scientific Director, Institute for Circumpolar Health Research, Yellowknife, Canada

*Panelists*

Elder Francois Paulette, Chair of Stanton Territorial Health Authority Elders Council, Fort Smith, Canada

Dr. Siv Kvernmo, Professor of Child and Adolescent Psychiatry, Department of Clinical Medicine, University of Tromsø, Tromsø, Norway

Ms. Donna Galbreath, Medical Director of Quality Assurance. Southcentral Foundation. Anchorage, Alaska, United States

*Jury findings and discussion*

Dr. Peter Bjerregaard, Professor of Arctic Health, Copenhagen, Denmark

*Closing remarks*

Dr. Howard Hu, Director and Professor, Dalla Lana School of Public Health, University of Toronto, Toronto, Canada

*Rapporteurs*

Jessica Bytautas

Anthea Darychuk

Jury members

Dr. Peter Bjerregaard, Professor of Arctic Health, Centre for Health Research, Greenland

Ms. Natalie Chafe-Yuan, Director of Policy, Planning and Evaluation, Department of Health and Social Services, Government of Nunavut

Dr. Malcolm King, Director of the Institute for Aboriginal Peoples’ Health, Canadian Institutes of Health Research

Dr. Michael Jong, Vice President of Medical Services, Labrador-Grenfell Health

Dr. Jeremy Veillard, Vice President of Research and Analysis, Canadian Institute for Health Information

Dr. Maureen Baikie, Chief Medical Officer of Health, Nunavut

